# Retroperitoneoscopic lumbar sympathectomy for the treatment of primary plantar hyperhidrosis

**DOI:** 10.1186/s12893-021-01393-y

**Published:** 2021-11-12

**Authors:** Kyung Jae Hur, Hyong Woo Moon, Yong Hyun Park, Woong Jin Bae, Hyuk Jin Cho, U-Syn Ha, Ji Youl Lee, Sae Woong Kim, Sung-Hoo Hong

**Affiliations:** 1 Department of Urology, Seoul St. Mary’s Hospital, College of Medicine, The Catholic University of Korea, Republic of Korea 222, Banpo-daero, Seocho-gu, Seoul, 06591 Republic of Korea; 2 Department of Urology, Fatima Hospital, Daegu, Korea

**Keywords:** Primary plantar hyperhidrosis, Retroperitoneoscopic lumbar sympathectomy, Compensatory sweating, Infrared thermography

## Abstract

**Background:**

Primary plantar hyperhidrosis (PPH) is an idiopathic disease, characterized by excessive sweating of the feet. It leads to significant disturbance in private and professional daily lifestyle, due to excessive sweating. The aim of this study is to present the safety, efficacy and procedures of retroperitoneoscopic lumbar sympathectomy (RLS) for treatment of PPH.

**Methods:**

RLS was performed 60 times in 30 patients (18 men, 12 women) with PPH in our institution from May 2019 to October 2020. All procedures were carried out by laparoscopy with retroperitoneal approach. Clinical data including patient demographics and perioperative, postoperative outcomes were evaluated. Recurrence of symptoms, and any adverse effects of surgery were evaluated after 7 to 30 days in outpatient clinic, and thereafter every 6 months.

**Results:**

Mean age of patients was 33.6 (± standard deviation 10.8) years. Fourteen and fifteen patients were previously treated with medical therapy or endoscopic thoracic sympathectomy (ETS) respectively. Mean preoperative quality of life (QoL) score of patients was 91.8 (VERY BAD), but postoperative 12 months (QoL) score decreased to 29.1 (MUCH BETTER). There was no serious postoperative complication. During the mean 22 months of follow-up period, no compensatory sweating was observed.

**Conclusions:**

RLS can be a safe and effective surgical treatment for severe PPH, especially for the patients with persistent plantar sweating even after conservative management and ETS. RLS also could be offered to surgeons who are familiar with retroperitoneal space anatomy as feasible surgical treatment for PPH.

## Background

Primary plantar hyperhidrosis (PPH) is characterized by excessive sweating of the feet. The etiology of the disease is not fully understood yet, but stimulation of sympathetic nerve seems to activate sweating from eccrine sweat gland. The onset of disease is usually young age. PPH is known to have a genetic factor, and its estimated prevalence is 1–16% in the general population [1, 2]. In many cases, excessive secretion of sweat from the hands and feet causes moderate to severe disturbance in daily social life. Also, patients often present with bromhidrosis, medical condition caused by the bacterial decomposition of sweat, which results in an unpleasant sweat odor. These problems can lead to significant impairment of the quality of life (QoL).

Conservative treatments are used in dermatology department, which consist of the application of anticholinergics, aluminum chloride solutions, iontophoresis, and subdermal injection of botulinum toxin [3–5]. However, these therapies have disadvantages of short-term effects and high rates of recurrence [6, 7]. For hands, if medical therapies are not satisfactory, endoscopic thoracic sympathectomy (ETS) is available for the surgical treatment of primary hyperhidrosis [8, 9]. Excessive sweating of the hands can be eliminated with this procedure in more than 90% of the patients. However, compensatory sweating and plantar hyperhidrosis remains unchanged after ETS in up to more than 50% of patients [9, 10].

In plantar hyperhidrosis, lumbar sympathectomy can be an effective surgical treatment for eliminating excessive sweating of the feet. However, there are not many reports regarding the safety and efficacy of lumbar sympathectomy for plantar hyperhidrosis in Asian countries.

Although thoracic sympathectomy has been introduced as surgical treatment for palmar hyperhidrosis and numerously reported with systematic investigations, lumbar sympathectomy for plantar hyperhidrosis has become clinically utilized only until recently due to previous worries of postoperative complications such as compensatory sweating and retrograde ejaculation [10, 11]. Although high postoperative patient satisfaction has been proven in general surgery department of European groups [12–14], the outcome of lumbar sympathectomy has not been reported much in Asian groups.

The objective of this study is to present the safety, efficacy and operation procedures of retroperitoneoscopic lumbar sympathectomy (RLS) in Asian groups.

## Materials and methods

### Patient selection

Prospective study of all patients who underwent RLS in our department from May 2019 to October 2020 was performed. All patients were referred from chest surgery department, due to compensatory plantar sweating which persisted after medical treatment or endoscopic thoracic sympathectomy. Medical records including patients’ demographics and previous medical history were evaluated. Preoperative and postoperative plantar sweating symptom and quality of life were evaluated using the standardized hyperhidrosis quality of life (QoL) questionnaire developed by de Campos et al. [15] in outpatient clinic, after 1 month and thereafter every 6 months after surgery.

### Postoperative outcomes evaluation

Preoperative and postoperative plantar skin temperature were measured using infrared thermal camera (FLIR C5™, FLIR® Systems, Inc.) to check the change of plantar skin temperature. Postoperative skin temperature was measured on the day after surgery, and every 6 months thereafter in the clinic.

### Operation procedure

**Fig. 1:**
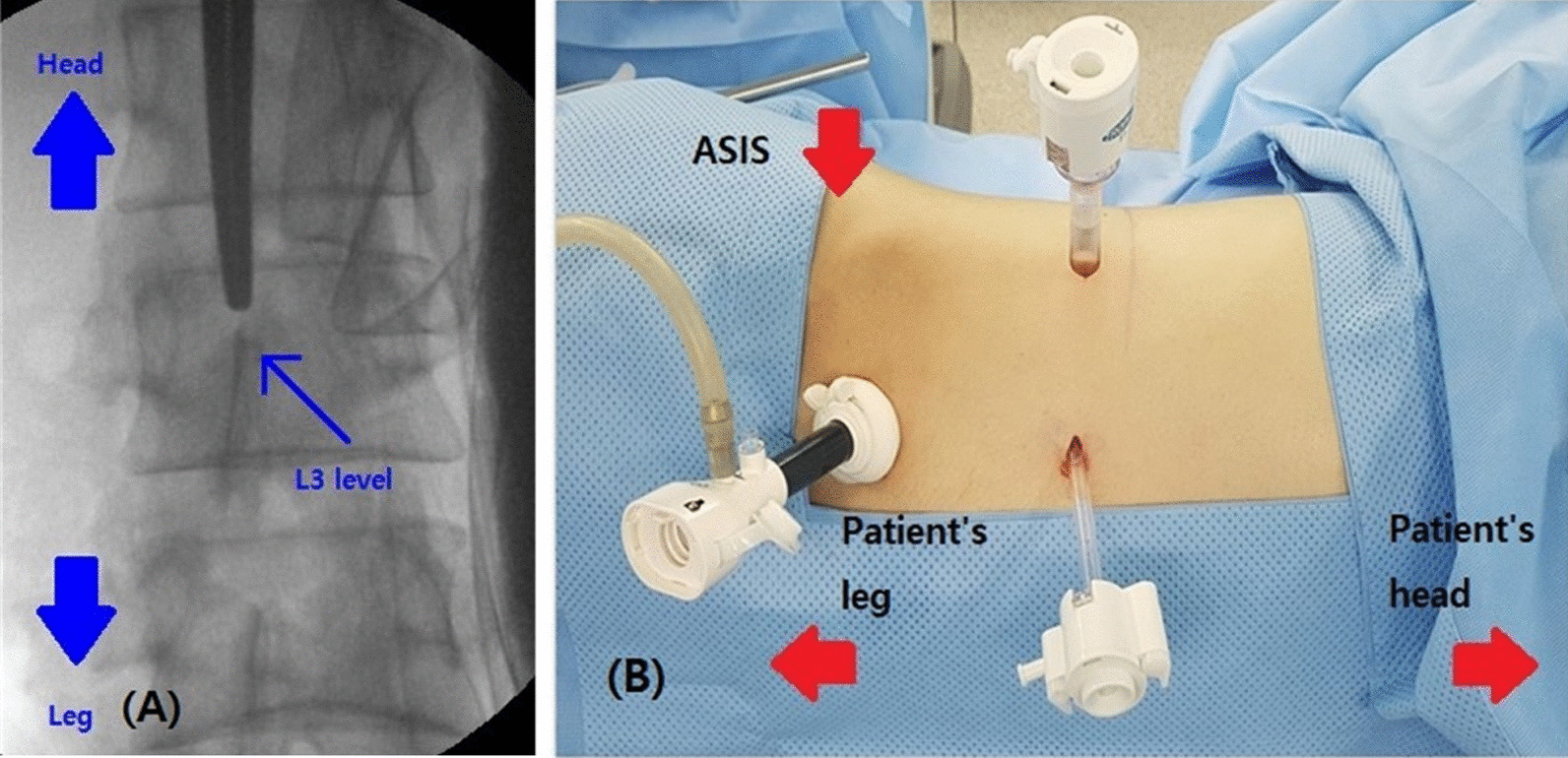
**A** L3 level marking under C-arm fluoroscopic view. **B** Port configuration. 1st camera port (balloon trocar) was inserted at medial and superior from the anterior superior iliac spine (ASIS). 2nd port was inserted at the middle clavicular line between the iliac crest and lowest rib. 3rd port was placed at the posterior axillary line

**Fig. 2:**
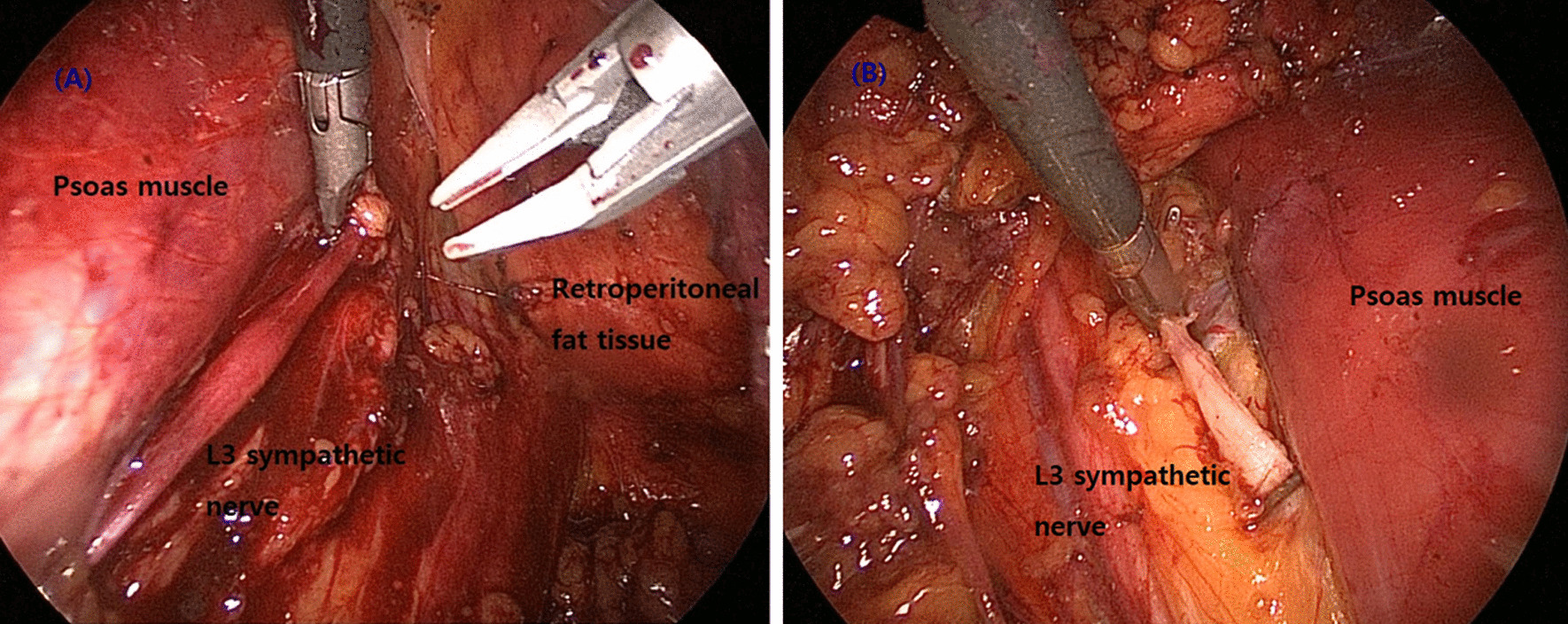
**A** Exposure of L3 sympathetic nerve during RLS. Sympathetic nerve is identified in the medial aspect of the psoas muscle. On the right side, the sympathetic chain lies posterior to the inferior vena cava. **B** On the left side, the sympathetic chain lies posterior and lateral to the infra-renal aorta

All operations were performed bilaterally under general endotracheal anesthesia. Patient is placed in lateral position. After hyperflexion of the table, we marked the projection of the third lumbar spine level onto the flank skin of the patient under a C-arm fluoroscopic view (Fig. 1A). After routine draping, about 1 cm length of skin incision was made at 2 cm medial and superior spot of anterior superior iliac spine. After skin incision, external oblique muscle, internal oblique muscle, and transveralis abdominis were dissected consecutively with splitting using hemostat. The transversalis abdominis was separated from the peritoneum. Retroperitoneal space was opened with blunt finger dissection. During this procedure, we made sure that there is no peritoneal opening. The round shape balloon device (AutoSuture ™, COVIDIEN) was inserted through the incision site to be ballooned up to 500 cc, for dilating retroperitoneal space. A 10 mm blunt tip balloon trocar was inserted into dilated space and ballooned with 30 cc of air. CO_2_ gas was insufflated to make pneumoretroperitoneum upto 15 mmHg. Camera device was inserted to the first trocar. We used two additional ports. 2nd port (5 mm) was inserted at the middle clavicular line between the iliac crest and rib. 3rd port (11 mm) was placed at the posterior axillary line at the same level (Fig. 1B). Initially, we confirmed the identification of L3 level with C-arm view after dissection of L3 nerve. But later on some learning experiences, we inserted 2nd and 3rd trocars at the L3 level which was identified by the C-arm view, and approached vertically to find the L3 nerve directly. After exposure of the retroperitoneal space, Gerota’s fascia was dissected. On the right side, the sympathetic chain lies posterior to the inferior vena cava, which was retracted anteriorly (Fig. 2A). On the left side, the sympathetic chain lies posterior and lateral to the infra-renal aorta. Pushing all structures anteriorly, retroperitoneal fat tissue was dissected. The sympathetic nerve was identified in the medial aspect of the psoas muscle (Fig. 2B). Both upper and lower 2 cm margin of sympathetic nerve were clipped using titanium clip (Ligaforce), and the segment was resected with metzembaum scissor. After position change to contralateral side, same method was done. Muscle layer and subcutaneous tissue were closed layer by layer.

### Statistical analysis

Data were analyzed using IBM SPSS version 24.0 (IBM, Chicago, Illinois, USA). Values are presented as mean ± standard deviation. Continuous variables of groups were compared using paired t-tests. Multiple linear regressions were used to determine the influencing factors of postoperative patient’s satisfaction and QoL score. P-value was considered statistically significant if less than 0.05.

### Ethical considerations

This study was approved by the Institutional Review Board of Seoul saint Mary’s hospital, the Catholic university college of medicine (Ethics approval number: KC20RISI0086) in accordance with the recommendations of the Helsinki Declaration. Informed consents were obtained from all the participants enrolled in the study.

## Results

### Patients’ demographics

RLS was performed 60 times in total of 30 patients (18 men, 12 women) in our institution from May 2019 to October 2020 by single surgeon. Of the 30 patients, their mean age was 33.6 (years) ranged from 18 to 66. Twenty two patients (73.3%) were young age, less than 40 years old. Mean BMI (Body mass index) was 22.9. Among them, 14 patients (43.7%) had medical therapies before, while 15 patients (46.8%) underwent ETS previously. Three patients (10%) had past history of percutaneous alcohol injection therapy, but plantar hyperhidrosis persisted. Patients’ demographics and characteristics are summarized in Table 1. Among all of the enrolled patients, eleven patients underwent simultaneous RLS and ETS with the chest surgery department, due to compensatory sweating of the trunk and feet. Nine patients underwent RLS alone.

**Table 1:** Patients’ characteristics and demographic data

	Number of patients (%)
Age (year)	
Mean (± SD)	33.6 (± 10.8)
< 20	2 (6.6%)
21–30	11 (36.6%)
31–40	9 (30.0%)
> 40	8 (26.6%)
Gender ratio (Male: female)	18:12
Mean BMI (± SD)	22.9 (± 2.8)
Previous therapy (Number of patients, percentage)	
Medical therapies (glycopyrrolate, aluminium chloride)	14 (43.7%)
Previous ETS	15 (46.8%)
Percutaneous alcohol injection	3 (9.3%)

*SD* standard deviation, *BMI* body mass index, *ETS* Endoscopic thoracic sympathectomy

### Perioperative outcomes

All procedures were carried out by laparoscopic surgery with retroperitoneal approach. There was no conversion to an open surgery. Mean operation time was 65 min including position change. Operation time decreased over learning experiences. Mean estimated blood loss was 18 ml, which was minimal. Twenty five patients (83.3%) discharged from the hospital within 24 h postoperatively. Seven patients (16.7%) discharged the day after surgery due to moderate wound pain, but no other specific postoperative complication was observed.

### Postoperative sweating symptom measurement

All patients experienced immediate improvement of plantar sweating postoperatively. Mean preoperative quality of life (QoL) score of patients was 91.8 (QoL score, 20; Excellent ~ 100; very bad), but late postoperative (12 months) QoL score decreased to 29.1 (Improved evolution of quality of life (QV) score, 20: Much better ~ 100; Much worse) (Table 2). One case of postoperative priapism occurred, but it resolved spontaneously.

**Table 2:** Comparison of QoL score between male and female Group

Variables	Male (Mean ± SD)	Female (Mean ± SD)	t	P
Preoperative QoL score	90.2 (± 2.5)	92.1 (± 1.9)	− 2.279	0.015
Late postoperative QoL score	30.9 (± 1.9)	25.9 (± 2.3)	6.276	< 0.001

*QoL* Quality of life

**Fig. 3:**
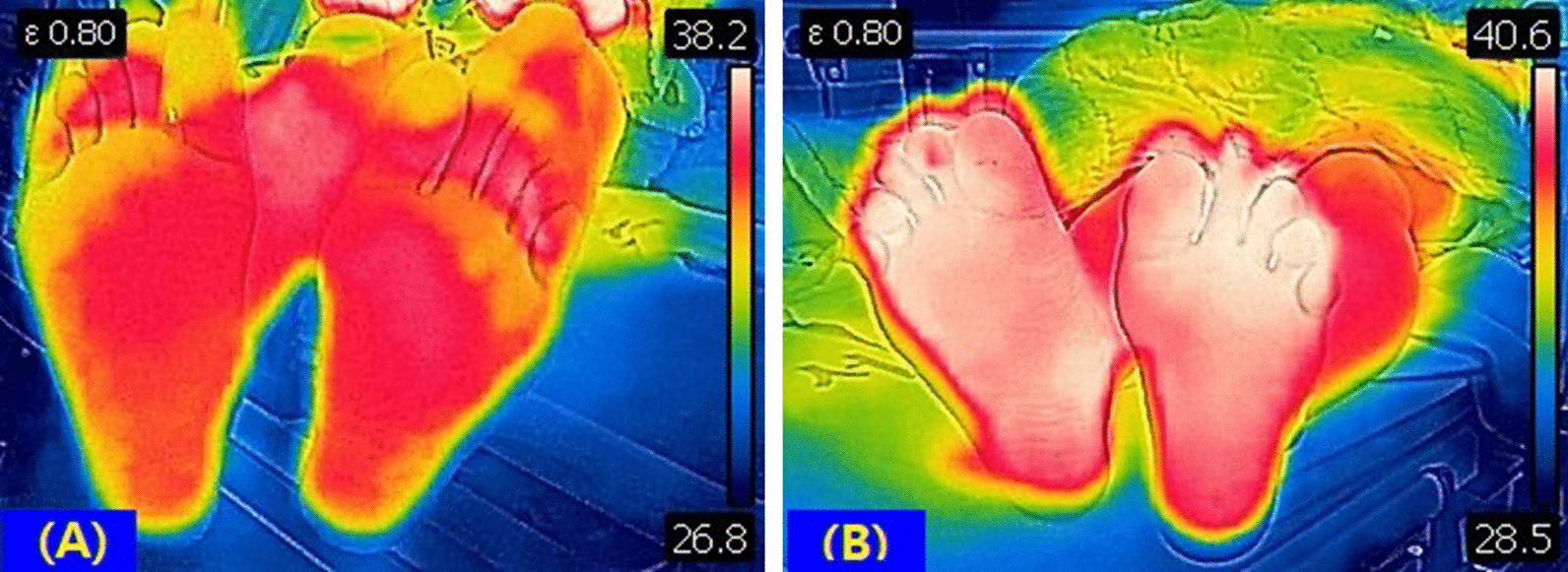
Comparison between preoperative & postoperative plantar skin temperature using infrared thermal camera. Postoperative plantar skin temperature **B** increased compared with preoperative one **A** due to decreased plantar perspiration after sympathectomy (Overall plantar temperature: 36.4 °C  → 38.5 °C, highest temperature: 38.2 °C  → 40.6 °C, lowest temperature: 26.8 ℃ → 28.5 °C)

Also, we confirmed increase of postoperative plantar skin temperature compared with preoperative one, due to decreased perspiration of plantar after sympathectomy. We used infrared thermal camera (FLIR C5™, FLIR® Systems, Inc.) to measure skin temperature (Fig. 3A, 3B).

Patient’s gender was significant factor in postoperative QoL score. However, age and body mass index (BMI) did not affect QoL score. Also, female group showed more higher QoL score than male group both preoperative and postoperatively (Tables 2, 3).

**Table 3:** Factors affecting late postoperative QoL score

Variables	β	t	P value
Gender	− 0.731	− 5.748	< 0.001
Age (years)	− 0.039	− 0.304	0.764
BMI	0.167	1.301	0.205

*BMI* body mass index. *95% CI* confidence interval

## Discussion

Primary focal hyperhidrosis is defined as excessive sweating from focal parts of human body. Common involved sites are face, soles, axilla and inguinal region. Primary hyperhidrosis is not caused by other underlying conditions. Secondary hyperhidrosis may be focal or generalized, caused by underlying medical disease or medicine use [1, 2]. Disease onset is usually from childhood to adolescent. The disease is not life threatening, however, bromhidrosis can cause bad smells in the involved area. It can cause excessive sweating from hands and feet, and interfere with quality of life.

The first line treatment is conservative therapies, such as anticholinergics, topical use of aluminum chloride solutions and iontophoresis. They can improve sweating symptoms as long as the methods are used, but require continuous use, which does not occur in most patients. The intradermal injection of botulinum toxin, which were used successfully for axillary hyperhidrosis, also can be tried [3–5]. However, injections into the sole of the foot are very painful and local anesthesia to hands and feet is difficult to be induced by the application of anesthetic cream. In addition, the effect of botulinum toxin is limited to a few months [6]. Percutaneous alcohol injection is also applied to treat PPH. It shows good responses after treatment, but also has disadvantages of moderate rates of recurrence, and complications that include complex regional pain syndromes as well [7].

Permanent cure can be achieved by surgical treatment. Endoscopic thoracic sympathectomy (ETS) is available as surgical treatment for primary palmar hyperhidrosis. ETS is effective for treating palmar sweating in more than 90% of the patients [8, 9, 16, 17]. However, plantar hyperhidrosis and compensatory sweating remains even after ETS in up to 28–70% of the patients [18, 19].

In plantar hyperhidrosis that is persistent even after ETS, endoscopic lumbar sympathectomy (ELS) can be an effective surgical treatment for eliminating plantar hyperhidrosis. ELS has been increasingly accepted for patients with severe plantar hyperhidrosis over the last few years in European countries. Rieger et al. have performed extensive experiences on the management of plantar hyperhidrosis using ELS. They reported the cohort of 178 ELS in 90 patients from December 2004 and September 2008, including 53 cases who had coexisting palmar hyperhidrosis and 24 cases undergone previous ETS. Follow-up after 3–45 months, hyperhidrosis was eliminated in 97% of patients, with recurrence of 3%. Compensatory sweating occurred in 44% of patients, and post-sympathetic neuralgia occurred in 42%. Overall 96% were satisfied with the result, and 92% replied that they will repeat the procedure if required [21–23].

Lima SO, et al*.* reported long-term results of RLS. Lumbar sympathectomy was performed 116 times in 58 patients (36 women and 22 men) from October 2005 to October 2014. Among 58 cases, 49 patients had fully answered the follow-up questionnaire in the clinic at a minimum of 12 months after RLS. Improvement in QoL due to symptom resolution was reported in 98% of the 49 patients. Mild to moderate increase in pre-existing compensatory sweating was noted. Three patients (5.2%) reported transient thigh neuralgia, and 19 (32.7%) reported transient paresthesia in the lower limbs, with no reports of retrograde ejaculation [24]. However, performance of ELS has not been reported in urology department, especially in Asian group yet.

Compensatory sweating is the main reason for dissatisfaction after ETS. Severe compensatory sweating after ETS can occur in about 3–5% of patients [11]. After lumbar sympathectomy alone, however, severe compensatory sweating was rare [22]. In our study, 11 patients underwent simultaneous ETS and RLS. Plantar hyperhidrosis was improved immediately after surgery.

Potential sexual dysfunction is worrisome postoperative complication in lumbar sympathectomy in male patients [20, 25]. Erection is mainly controlled by parasympathetic nerves, whereas ejaculation is controlled by sympathetic nerve stimulation. The lowest thoracic (T10) and first lumbar (L1) ganglia play key roles in ejaculation [26]. In similar studies, Rieger et al. reported ejaculation disorders after lumbar sympathectomy in 6–54% of the men, but were usually observed when the upper lumbar ganglion was resected in the sympathectomy, especially on both sides [22, 23]. The incidence of ejaculation disorders after RLS is low, which indicates that permanent sexual function disorders are not common after lumbar sympathectomy at the level of the third or fourth lumbar vertebral body with resection of the sympathetic trunk.

Accordingly, adverse effects related to sexual function after lumbar sympathectomy should be informed to male patients before surgery. Although, these adverse effects are not common, and self- limiting according to up to-date postoperative reports.

Theoretically, secretion function from the eccrine glands is controlled by the sympathetic nervous system. The preganglionic neurons responsible for the innervation of the eccrine sweat glands in the feet originate from the lateral horn of the lower thoracic and upper lumbar spinal cord. The nerve fibers go through the white ramus communicans to the sympathetic trunk where they run caudally in interganglionic branches, and are switched to postganglionic neurons at levels of the sympathetic trunk ganglia. Postganglionic neurons travel through the grey rami communicans to spinal nerves L4 to S3, over which they arrive in the peripheral portion of sweat glands of the feet. Therefore, resection of the sympathetic trunk from the upper segment of the third lumbar vertebral body to the lower edge of the fourth lumbar vertebral body results in blocking of the sympathetic conduction to the spinal nerves L4–S3, thereby eliminating sweat secretion of the feet [27, 28].

Determining the appropriate extent of sympathetic ganglion resection is questionable. Cheng A, et al. reported the recurrence rate of compensatory sweating after sympathectomy. The results varied according to the type of sympathectomy and extent of nerve resection [29]. Persistent skin moisture was more common after the resection of an interganglionic resection reported in previously published studies. We mainly resected ganglionic segments of the lumbar sympathetic nerve, and complete anhidrosis was not common. Most patients showed moderate improvement in sweating postoperatively with satisfactory quality of life.

Retroperitoneal approach facilitated efficient identification of the lumbar sympathetic chain for urologists, who are familiar with retroperitoneal organ surgery such as kidney, ureter and bladder. Psoas muscle, which is an important landmark to find sympathetic nerve chain, is one of the structures composing retroperitoneal space. Adjacent structures, including ureter, inferior vena cava and Gerota’s fascia also compose retroperitoneal space anatomy. Another advantage of our approach is that the port placement at the L3 level provided accurate identification of sympathetic ganglion and direct approach to it. This method also resulted in reducing unnecessary adjacent tissue damage.

Also, minimally invasive retroperitoneoscopic approach resulted in favorable perioperative and postoperative outcomes, including minimal blood loss, early mobilization and start of oral intake of patient. All patients discharged from the hospital within 24–48 h postoperatively. Postoperative wound pain was minimal because muscle layers were dissected with hemostat, not incised. None of our patients required intravenous analgesia postoperatively.

Additionally, infrared thermography is based on the emission of infrared radiation from a certain object or region as projection through an infrared thermal camera [30]. This instrument enables to detect the heat distribution on the object’s surface, and at the same time to measure peripheral temperature. Researches with the use of infrared thermography have been reported in the various medical fields, including musculoskeletal & vascular departments, obstetrics, gynecology, and nephrology [31–34]. We applied infrared thermography for measuring the differences between preoperative & postoperative plantar temperature to strengthen our results. Infrared thermography can be non-invasive and objective tool to evaluate the efficacy of sympathectomy, because decreased perspiration from plantar will lead to increase of skin temperature.

The optimal change of plantar temperature after sympathectomy is not strictly settled yet, but it is notable that most patients showed satisfactory symptom score in the clinic with increase of plantar skin temperature during the follow up period.

Several limitations are present in our study. First, the numbers of enrolled patients is small, and postoperative follow up period is intermediate. Second, the standard of optimal change of plantar temperature after sympathectomy is not fully established yet. Further studies with larger numbers of cases and long term postoperative follow-up will be required. Although retroperitoneoscopic lumbar sympathectomy was performed in the urology department, it could be applied in other general surgery department, because retroperitoneal approach could facilitate operation for urologists and other surgeons who are familiar with retroperitoneal surgery.

## Conclusions

RLS can be a safe and effective surgical treatment for PPH, especially for patients who do not respond to medical treatment or persist plantar sweating after ETS.

## Data Availability

The data that support the findings of this study are available from the corresponding author on reasonable request.

## Abbreviations

PPH: Primary plantar hyperhidrosis
RLS: Retroperitoneoscopic lumbar sympathectomy
ETS: Endoscopic thoracic sympathectomy
BMI: Body mass index
